# miRNA-214-5p inhibits prostate cancer cell proliferation by targeting SOX4

**DOI:** 10.1186/s12957-021-02449-2

**Published:** 2021-12-04

**Authors:** Guangchi Xu, Yin Meng, Lihe Wang, Bo Dong, Feifei Peng, Songtao Liu, Shukui Li, Tao Liu

**Affiliations:** Department of Urological Surgery, The Second Affiliated Hospital of Qiqihar Medical University, No. 37 Zhonghua West Road, Jianhua District, Qiqihar, 161000 Heilongjiang Province China

**Keywords:** Prostate cancer, miRNA-214-5p, SOX4

## Abstract

**Background:**

Prostate cancer is the most common malignant tumor in men. Due to the lack of theoretical research on its pathogenic mechanism, the current cure rate is still low. miRNAs play an important role in the pathogenesis of various cancers. miRNA-214-5p plays an important role in the development of a variety of cancers. This study aims to explore the expression level of miR-214-5p in prostate cancer and make a preliminary study of its molecular mechanism in the development of prostate cancer to provide effective new strategies for the treatment of prostate cancer.

**Methods:**

The target genes of miRNA-214-5p were predicted with bioinformatics technology, and the target relationship between miRNA-214-5p and its target genes was verified with dual luciferase reporter assay. RT-qPCR and Western blot were used to detect the expression levels of miRNA-214-5p and target genes in 50 clinical samples and two common prostate continuous cell lines, respectively. The targeting relationship between miRNA-214-5p and its target genes was verified with clinical data. miRNA-214-5p and miRNA-214-5p inhibitor was over-expressed in DU-145 cell lines to verify the effect of miRNA-214-5p on prostate cancer cell proliferation and SOX4 gene expression. And the mechanism of miRNA-214-5p inhibiting the proliferation of prostate cancer cells were analyzed by detecting the expression difference of downstream factors of SOX4 pathway. Bioinformatics analysis showed that miRNA-214-5p combined with SOX4 3′UTR region, and dual luciferase reporter assay further verified the reliability of the predicted results. The low expression of miRNA-214-5p was observed in prostate cancer tissues and cells, while high expression of SOX4 was observed in prostate cancer tissues and cells.

**Results:**

Overexpression of miRNA-214-5p to prostate cancer cells significantly inhibited the proliferation of cancer cells, and the expression of SOX4 was inhibited in the transfected cell line. After transfection of miRNA-214-5p inhibitor into prostate cancer cells, the cell proliferation rate further increased. Meanwhile, overexpression of miRNA-214-5p effectively inhibited the expression of SOX4 downstream factors, including c-Myc, eIF4E, and CDK4. However, the specific knockdown of SOX4 through SOX4 shRNA significantly reduced the proliferation of prostate cancer cell lines.

**Conclusions:**

miRNA-214-5 can inhibit the proliferation of prostate cancer cells by specifically targeting S0X4 and inhibiting the expression of growth factors downstream of this pathway.

## Abstract (article highlights)

1. Low expression of miRNA-214-5p is observed in prostate cancer cells.

2. miRNA-214-5p inhibits the proliferation of prostate cancer cells in vitro by targeting SOX4.

## Background

For male population, prostate cancer is most common malignant tumor second to lung cancer [1, 2] and also the fifth lethal disease in the world [3]. According to statistics, a total of 1,276,106 prostate cancer patients were newly diagnosed in 2018, and 358,989 of them died, accounting for 3.8% of the deaths of male cancer patients [4].

Studies have shown that early prostate cancer can be accurately detected by prostate specific antigen (PSA) screening. Under ideal circumstances, PSA screening can slightly reduce the mortality of prostate cancer patients with specific diseases within 10 years, but it has no significant effect on the final survival rate [5]. Some other studies have pointed out that if patient’s life expectancy cannot be effectively extended, PSA screening can cause unnecessary side effects such as tissue biopsy complications. It leads to overdiagnosis and overtreatment, reduces the quality of life of patients, increases personal and national medical expenses, and reduces the value of PSA and digital rectal examination (DRE) as early detection methods [6].

In recent years, the clinical usability of integrated positron emission tomography (PET) and magnetic resonance imaging (MRI) scanners has enabled multimodal, combined metabolic receptor, anatomical, and functional imaging joint assessment programs to be applied in practice [7]. Based on the available data, we have found that PET/MRI has very significant application value in the following aspects, including diagnosis of primary early prostate cancer; assistance of tissue biopsy positioning; predicting or detecting the aggressiveness of tumors in the active surveillance phase; early diagnosis of recurrent prostate cancer; and credible guidance for the clinical treatment of prostate cancer [8]. But at the same time, we should also recognize the shortcomings of the combined PET/MRI diagnosis technology, that is, the accuracy of this image-based diagnosis is very dependent on the interpretation of radiologists and urologists, which limits the large-scale promotion of this diagnostic technique [9]. Although the diagnostic technology of prostate cancer is constantly updated and iterated in modern society, the actual situation reminds us to have a deeper and more comprehensive understanding of the pathogenesis and development process of prostate cancer, so as to use effective therapeutic drugs to significantly improve the cure rate and survival rate of prostate cancer patients with the continuously optimized support of early diagnosis technology in the future.

MicroRNAs (miRNAs) are a kind of non-coding RNAs derived from endogenous transcripts. They can facilitate genome regulation and fine-tuning through post-transcriptional gene silencing [10]. Usually, these short RNAs are able to bind to specific sites in the 3′-UTR of their target genes and mediate mRNA degradation or block gene translation through complete or incomplete base pairing [11, 12]. As we all know, cancer is one of the most major causes of human death [13]. In recent years, more and more studies have found that miRNAs play an important role in tumorigenesis and tumor progression as they can participate in a variety of malignant behaviors related to cellular processes by targeting many transcripts [14]. According to previous reports, miRNAs play an important role in the development of breast cancer, lung cancer, colon cancer, ovarian cancer, gastric cancer, and prostate cancer [15]. In the research progress of prostate cancer, miRNA-200b and miRNA-200c have been reported to be closely related to the development of prostate cancer, and they may be used as prognostic markers of prostate cancer [16]. As it has been widely confirmed that miRNAs are involved in cancer regulation, researchers are eager to explore the possibility of using them as therapeutic targets and tools to reverse the abnormal expression of miRNAs to normal levels, delivering significant therapeutic intervention [17].

A large number of studies have confirmed that miRNA-214-5p plays an important role in a variety of tumors [18]. In the study of small or non-small cell lung cancer (NSCLC), researchers found that the expression of multiple miRNAs, including miRNA-214-5p in radiotherapy/chemotherapy resistant NSCLC cells was significantly higher than that in radiosensitive cells, and the high expression of miRNA-214-5p can significantly improve the apoptosis caused by radiotherapy [19, 20]. Another study on gastric cancer showed that miRNA-214-5p inhibited the tumor enhancement effect of tumor-associated fibroblast on gastric cancer by targeting fibroblast growth factor 9 (FGF9) in tumor-associated fibroblast and regulating epithelial to mesenchymal transition in gastric cancer [21]. Recent studies have shown that a long non-coding RNA-LINC00324 could competitively inhibit the function of miRNA-214-5 to inhibit the expression of cyclin-dependent protein kinase 6 (ICDK6), cyclin D1 (CCND1), murine double minute 2 (MDM2) and murine double minute 4 (MDM4), thus promoting the proliferation of immature ovarian teratoma cells and inhibiting their apoptosis [22]. In addition to the above studies, it was also reported that the expression of miRNA-214-5p in thyroid papilloma, liver cancer, and esophageal squamous cell carcinoma was also significantly down regulated, and overexpression of miRNA-214-5p could significantly promote the apoptosis of cancer cells, and inhibit the proliferation, migration, and invasion of cancer cells [23]. Moreover, miRNA-214-5p can affect the incidence and development of cancer by targeting a variety of tumor-related regulatory factors [24].

Relevant research data showed that in urine samples of prostate cancer patients, the expression level of miRNA-214 is significantly lower than that of non-cancer patients. At the same time, by evaluating the expression level of miRNA-214 in urine, it is possible to accurately distinguish prostate cancer patients from non-cancer patients. In the population, the sensitivity and specificity are as high as 89% and 80%, respectively [25]. Other studies have shown that there are significant differences in the transcription level of miRNA-214 in African American and Caucasian prostate cancer cell lines [26]. These results indicate that miRNA-214 may be involved in the development of prostate cancer, but the expression pattern of miRNA-214 in clinical samples of prostate cancer and the mechanism of its involvement in prostate cancer are not yet clear. In order to explore the pathogenic mechanism of miRNA-214 in the development of prostate cancer, this study tried to deeply explore the role of miRNA-214-5p in the proliferation of prostate cancer cells and its possible molecular mechanism to provide a potential new target for the clinical treatment of prostate cancer.

## Test method

### Instrument and reagent

DU-145 (ATCC HTB-81) and PC-3 (ATCC CRL-1435) cells were purchased from ATCC and the culture media were MEM medium and F12 medium (Catalog No.: 30-2003; 30-2004) specially made by ATCC. PrimeSTAR HS DNA Polymerase (Catalog No.: R010A) was purchased from Takara Bio (Dalian) Co., Ltd.. Psicheck-2 and Dual-Luciferase Reporter Assay System (Catalog No.: C8021; E1910) were purchased from Promega. Trizol (Catalog No.: 15596018) was purchased from Thermo Fisher. RIPA protein lysate and BCA protein concentration kit (Catalog No.: P0013B) were purchased from Beyotime Biotechnology Co., Ltd. Reverse transcription kit (Catalog No.: RR037B) was purchased from TAKARA Biological Company. Fluorescent quantitative PCR reagent (Catalog No.: 491385001) was purchased from Roche Company. SOX4, c-Myc, eIF4E specific antibodies (Catalog No.: sc-518016, sc-40, sc-100731) were purchased from Santa Cruz. HRP-labeled goat anti-mouse secondary antibody (Catalog No.: HS201-01) was purchased from TransGen Biotech Co., Ltd. X-tremeGENE siRNA Transfection Reagent (Catalog No.: 4476093001) was purchased from Roche. CCK-8 reagent (Catalog No.: C0037) was purchased from Beyotime Biotechnology Co., Ltd. 4-15% gradient SDS-PAGE adhesive (Catalog No.: 4561086) was purchased from Bio-Rad. Other inorganic reagents were purchased from Shaanxi Xilong Chemical Co., Ltd.

The full wavelength microplate reader (Model: 450 ) was purchased from Bio-Rad, USA. The small table type refrigerated centrifuge (Model: 75002456) was purchased from Thermo Fisher, USA. Western blot electrophoresis and transfer instrument (Model: 1645050) was purchased from Bio-Rad, USA. Ordinary PCR instrument (Model: T100) was purchased from Bio-Rad. Fluorescent quantitative PCR instrument (Model: QuantStudio 6) was purchased from Thermo Fisher. The chemiluminescence colorimeter (Model: 1708265) was purchased from Bio-Rad.

### Tissue collection

The tumor tissues and adjacent non-cancerous tissues (normal tissues) of prostate cancer patients were obtained during the surgery for prostate cancer patients from 2017 to 2019 in Urology Department of the Second Affiliated Hospital of Qiqihar Medical University. All obtained clinical tissues were rapidly frozen in liquid nitrogen and stored at − 80 °C. All tumor and normal tissue samples were confirmed pathologically before use [1]. The specific differential diagnosis criteria were as follows: the samples of tumor and normal tissues collected in the process of surgery were analyzed by magnetic resonance spectroscopy, and if the ratio of (choline + creatine)/citrate (CC / C) was higher than 0.99, the tissues were judged as prostate cancer tissues [27]. This test has been approved by the ethics committee of the Second Affiliated Hospital of Qiqihar Medical University (approval no. 2016-029).

### Prediction of miRNA-214-5p target genes

The binding site of SOX4 targeted by miRNA-214-5p was predicated on TargetScan (http://www.targetscan.org/vert_72/) [28], an online analysis website of microRNA. According to the guidance of online prediction website, miRNA-214-5p was input to predict the target site of SOX4.

### Luciferase reporter assay

According to the predicted results, the SOX4 3′-UTR promoter region was generated by the amplification of the results polymerase chain reaction (PCR), and it was recombined into the polyclonal site of psicheck2 dual luciferase reporter vector to construct the recombinant plasmid.

Dual luciferase activity assay: the dual luciferase reporter system of Promega Company was used to detect the activity of firefly luciferase and renilla luciferase. After HEK-293 T cells were rinsed with PBS, PLB (passive lysis buffer) was added into each well of the cell plate which was then placed on a plate oscillator to be shaken violently for 15 min at room temperature to make cells fully lytic. The cells were collected in a centrifuge tube, and centrifuged at 12,000*g* for 30 s. About 20 μL cell lysate supernatant was added into a 96-well ELISA plate, and then LAR II was added to each well to read the enzyme activity of firefly luciferase. After that, Stop & GLo reagent was added to read the enzyme activity of renilla luciferase. The relative luciferase activity of each sample was based on the ratio of the activity of renilla luciferase to that of firefly luciferase.

### Cell culture

Prostate cancer cell lines DU-145 and PC-3 were purchased from ATCC. The cell culture medium was RPMI 1640 medium supplemented with 10% fetal bovine serum and 1% penicillin/streptomycin. The cells were incubated in a thermostatic biochemical incubator with 5% CO_2_ at 37 °C.

### miRNA transfection experiment

miRNA was synthesized in Shanghai GenePharma Co., Ltd, and the transfection was performed strictly in accordance with the instructions of X-tremeGENE siRNA Transfection Reagent of Roche [29]. DU-145 and PC-3 were laid in a 24-well plate with a volume of 3 × 10^5^/well. The miRNA mimics and control miRNA were transfected into the cells, and then the protein and RNA expression level were detected with RT qPCR and Western blot.

### CCK-8 experiment

The proliferation efficiency of the transfected cells was measured at 6 h, 24 h, 48 h, and 72 h, respectively, as per the instructions [30]. About 50 μL CCK-8 solution was added to each well of the 24-well cell culture plate, and the well without cells were used as negative control. The cells were incubated in the cell culture chamber for 2 h, and the absorbance value at 450 nm was measured with the spectrophotometer.

### Detection of the expression of miRNA-214-5p and SOX4 in tissue samples

#### Detection of RNA level

Trizol method was used to extract total RNA from tissues, and TAKARA reverse transcription kit was used to reverse transcribe RNA into cDNA. According to the instructions of Roche fluorescent quantitative PCR reagent, RT-qPCR experiment was performed to detect the change of the relative expression levels of miRNA-214-5p, SOX4, and internal reference gene GAPDH mRNA. Related values were calculated with ΔΔCt method.

#### Detection of protein level

One hundred milligrams of tumor tissues and normal tissues were mixed with 1 mL RIPA lysate and placed on ice for 30 min after full homogenization. During this period, the samples were oscillated every 10 min, and then centrifuged at 4 °C with a speed of 12,000*g* × 10 min. The supernatant was collected, and the protein concentration was determined with BCA method. Five times of sample buffer was added to the total protein extracted from the tissue, and a 5-min boiling water bath was provided. About 10 μL protein sample were added to 4–15% pre-loaded SDS-APGE protein gel, and electrophoresis was made at constant pressure of 200 V for 40 min. Then, the protein gel was placed in the membrane transfer system at constant pressure of 120 V for 60 min. After the transfer printing, the protein gel was sealed in 5% BSA blocking solution for 1 h at room temperature. It was washed with PBS’T, SOX4, and c-Myc, and then eIF4E first antibody (1:1000 dilution) was added for incubation at room temperature for 1 h. After washing with PBS’T, HRP-labeled goat anti-mouse secondary antibody (1:5000 dilution) was added for incubation at room temperature for 1 h. And then, it was colored with ECL chemiluminescence solution in ChemiDoc XRS+ Gel Imaging System.

### SOX4 shRNA knockdown experiment

The shRNA targeting human SOX4 gene was designed on Invitrogen online shRNA website. The synthesized shRNA was cloned into the lentiviral vector pGhU6, and the correctness of the vector was verified with sanger sequencing. shRNA sequence: Forward 5′-aaccccgcgacaagatccctttcattcaagagatgaaagggatcttgtcgctttttgg aa-3′; Reverse 5′-tcgattccaaaaagcgacaagatccctttcatctcttgaatgaaagggatcttgtcgcggggtt- 3′.

Upon the construction of the vector, pGhU6 auxiliary plasmids pspAX2 and PMD2.G were co-transfected into HEK293T cells. After 60 h, the cell culture medium was collected to obtain the successfully packaged lentiviral solution. The specific knockdown of SOX4 can be induced by transducing the lentivirus into PU-145 and DC-3 cell lines.

### Data analysis

All statistical data were expressed as mean ± standard error (Mean ± SE). The differences between the experimental groups were analyzed with multiple comparison correction variances or t-test (Primer 5, GraphPad Software). *P* < 0.05 was considered as significant difference.

## Results

### Combination of miRNA-214-5p and SOX4 3’UTR regional specification

**Fig. 1:**
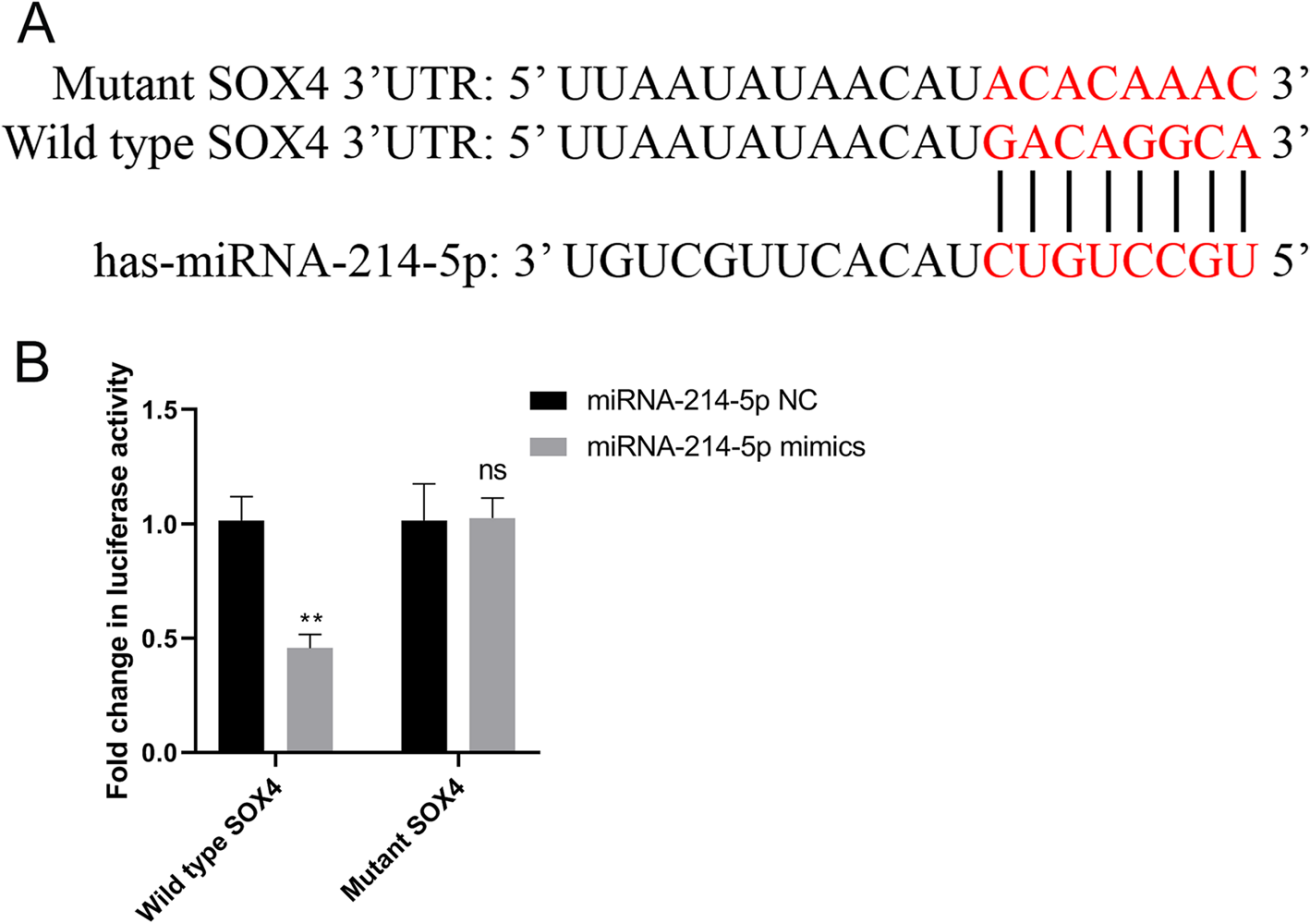
Regions of miRNA-214-5p specifically binding to SOX4 3′-UTR. Prediction of possible regions of miRNA-214-5p binding to SOX4 3′-UTR by bioinformatics website (**A**), and the specific binding of miRNA-214-5p to SOX4 3′-UTR was verified with dual luciferase reporter assay (**B**) miRNA negative control group was used as control (*P < 0.05, **P < 0.01)

In order to identify the target gene binding sites of miRNA-214-5p on SOX4 3′- UTR, the bioinformatics software was used to predict the potential target sites of miRNA-214-5p on human SOX4 gene 3′UTR. The predicted results showed that the sites from 2391 bp to 2398 bp on human SOX4 gene 3′UTR (Fig. 1A) are most probably able to bind to miRNA-214-5p. To further verify the accuracy of this prediction, the target gene was cloned into the dual luciferase reporter vector, psiCheck-2. The synthesized miRNA-214-5p mimics and negative control were co-transfected with the recombinant vector into HEK-293 T cells. The results showed that miRNA-214-5p mimics could bind to SOX4 3′-UTR target sequence, significantly reducing the relative expression of luciferase (Fig. 1B).

### miRNA-214-5p was lowly expressed and SOX4 was highly expressed in prostate cancer tissues

**Fig. 2:**
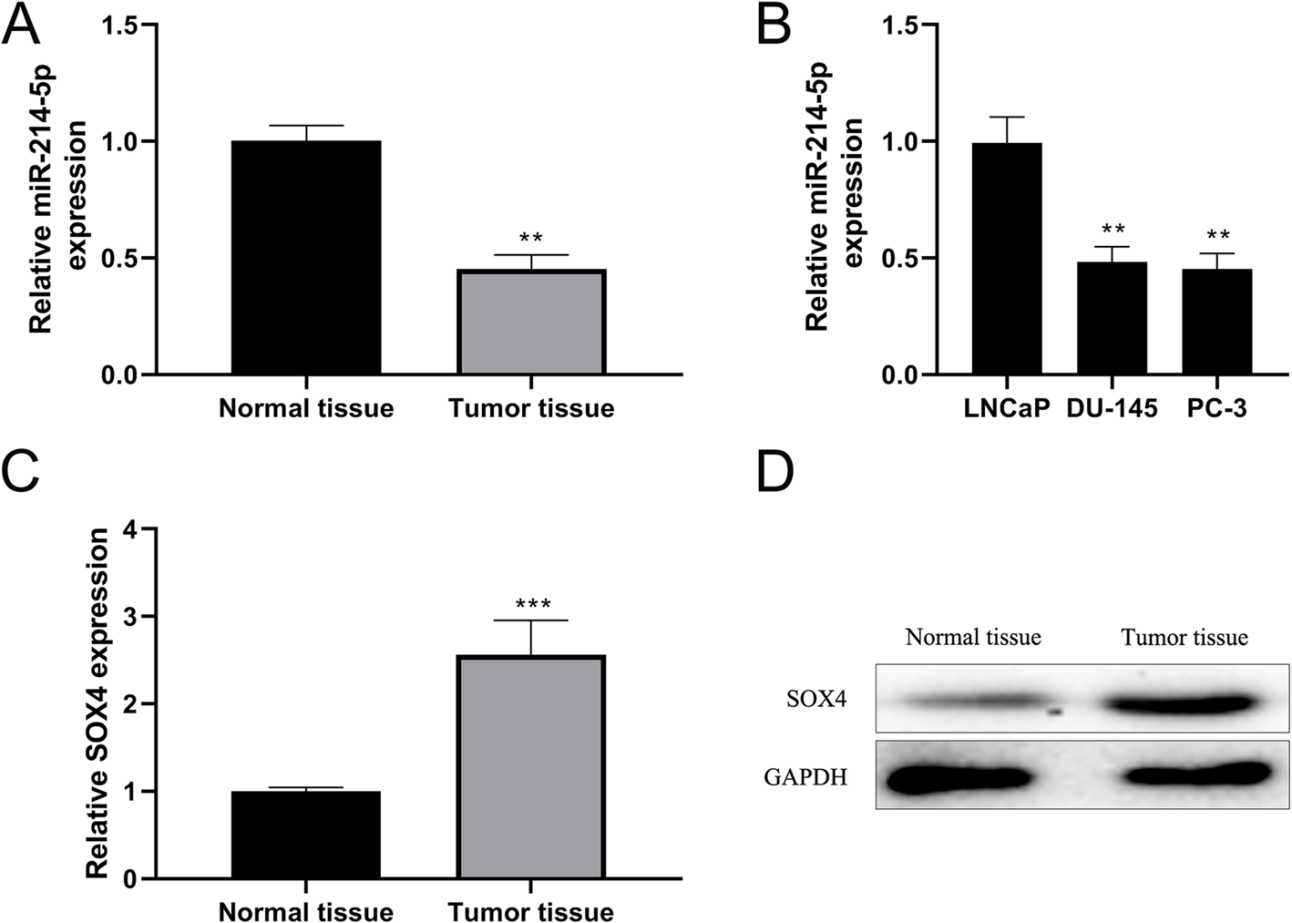
Expression patterns of miRNA-214-5p and SOX4 in prostate cancer tissues and cell lines. RT-qPCR assay was used to detect the expression of miRNA-214-5p transcription in prostate tumor tissues and normal tissues (**A**), and RT-qPCR assay was also used to detect the mRNA expression of miRNA-214-5p in prostate cancer cell lines DU-145 and PC-3 as well as control cell line LNCaP (**B**). Meanwhile, RT-qPCR (**C**) and Western blot (**D**) were used to detect the transcription and translation levels of SXO4 in prostate tumor and normal tissues, respectively. GAPDH was an internal reference protein. Normal tissues were used as control (*P < 0.05, **P < 0.01, ***P < 0.001)

According to the expression differences of miRNA-214-5p transcription in clinically collected prostate cancer tissue samples and normal tissue samples, it was found that the expression of miRNA-214-5p in prostate cancer tissues was significantly lower than that in normal tissues (Fig. 2A). In order to verify whether miRNA-214-5p has the same expression pattern in prostate cancer cell lines, we selected DU-145 and PC-3 cell lines. According to literature, most prostate cancer cell lines do not express endogenous androgen receptors, but researchers have detected the androgen receptor mRNA and protein in both DU-145 and PC-3 cell lines, which indicated that DU-145 and PC-3 cell lines are excellent prostate cancer model cell lines [31]. Similar results were observed in prostate cancer cell lines DU-145 and PC-3 (Fig. 2B). At the same time, the expression of SOX4 transcription and translation in prostate cancer tissues and normal tissues were explored. The results showed that mRNA and protein levels of SOX4 in prostate cancer tissues were significantly higher than those in normal tissues (Fig. 2C, D), indicating that SOX4 was highly expressed in prostate cancer tissues.

### Overexpression of miRNA-214-5p inhibits cell proliferation, overexpression of miRNA-214-5p inhibitor inhibits the proliferation efficiency of prostate cancer cell lines

**Fig. 3:**
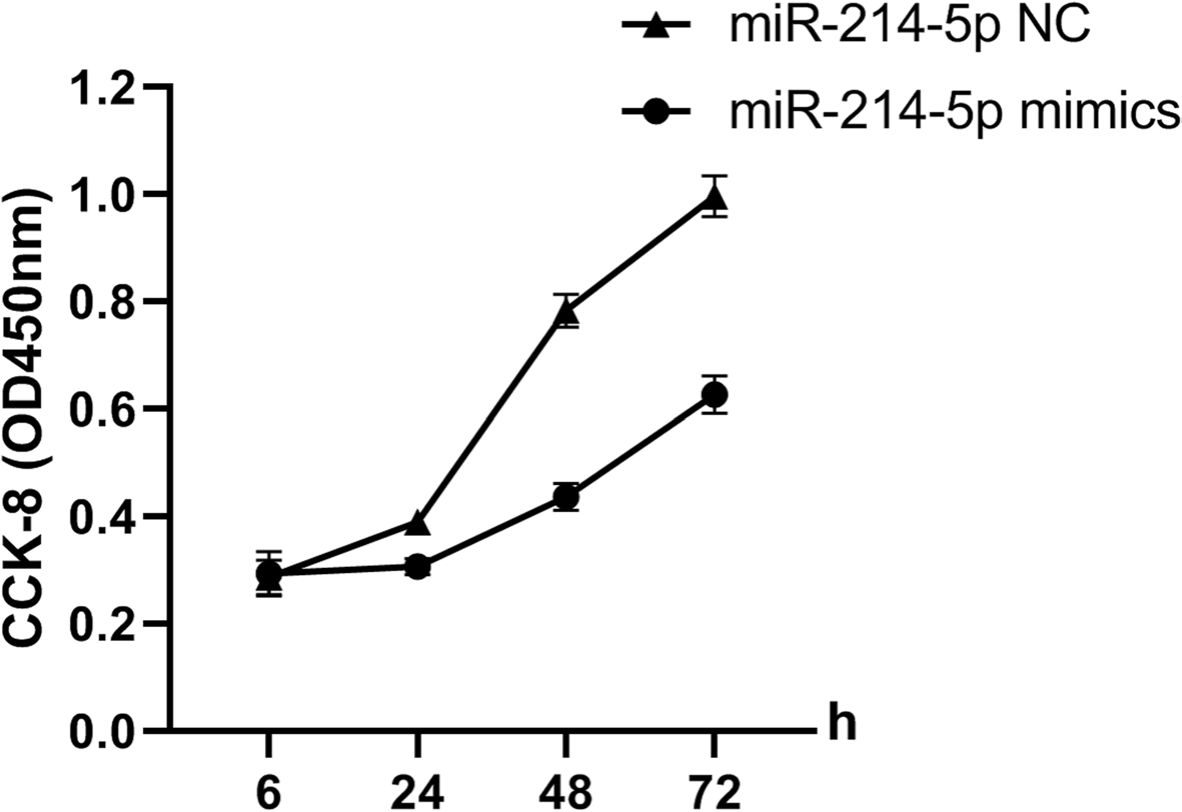
miRNA-214-5p inhibits cell proliferation. DU-145 cells were seeded into 24 wells, and miRNA-214-5p mimics, miRNA-214-5p inhibitor, and negative control were transfected into the cells, respectively. CCK-8 kit was used to detect cell proliferation at 6 h, 24 h, 48 h, and 72 h, respectively

The above results indicated that the expression of miRNA-214-5p in tumor tissue cells was decreased. Therefore, we wanted to explore the role of miRNA-214-5p in cell proliferation. To this end, the miRNA-214-5p mimics, miRNA-214-5p inhibitor and negative control were transfected into DU-145 cells, respectively, and the cell proliferation was detected with CCK-8 detection kit. The results showed that miRNA-214-5p mimics could significantly reduce the cell proliferation, and miRNA-214-5p inhibitor significantly increased cell proliferation efficiency (Fig. 3).

### miRNA-214-5p inhibited the expression of SOX4

**Fig. 4:**
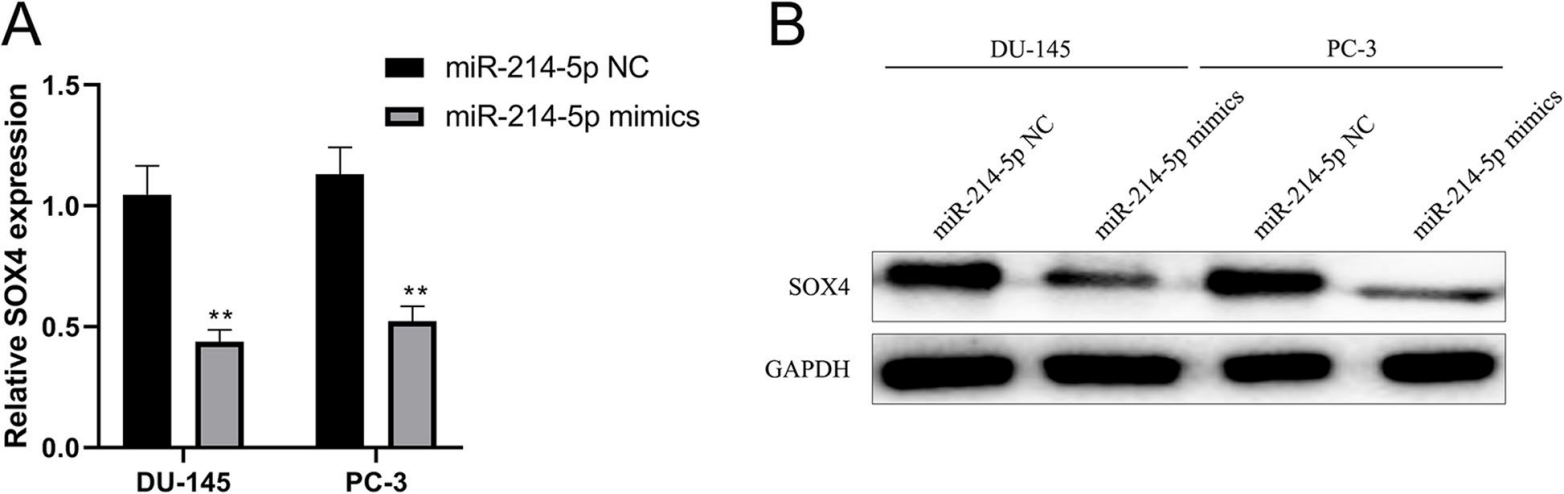
miRNA-214-5p inhibits the expression of SOX4. miRNA-214-5p mimics and negative control were transfected into DU-145 and PC-3 cells, respectively. After 48 h, total RNA and protein were extracted respectively. The transcription and translation levels of SOX4 were respectively measured with fluorescence quantitative PCR assay (**A**) and Western blot (**B**). miRNA control group was used as control (*P < 0.05, **P < 0.01)

The above results showed that the expression of miRNA-214-5p decreased in prostate cancer patients while that of SOX4 increased. In order to verify that miRNA-214-5p can directly lead to the expression of SOX4 in vitro, the change of SOX4 was measured by overexpression of miRNA-214-5p in vitro. The results showed that transfection of miRNA-214-5p mimics in prostate cancer cell lines DU-145 and PC-3 could significantly reduce the expression of SOX4 mRNA and protein (Fig. 4), indicating that miRNA-214-5p may directly affect SOX4 gene.

### miRNA-214-5p inhibited the expression of downstream factors of SOX4 pathway

**Fig. 5:**
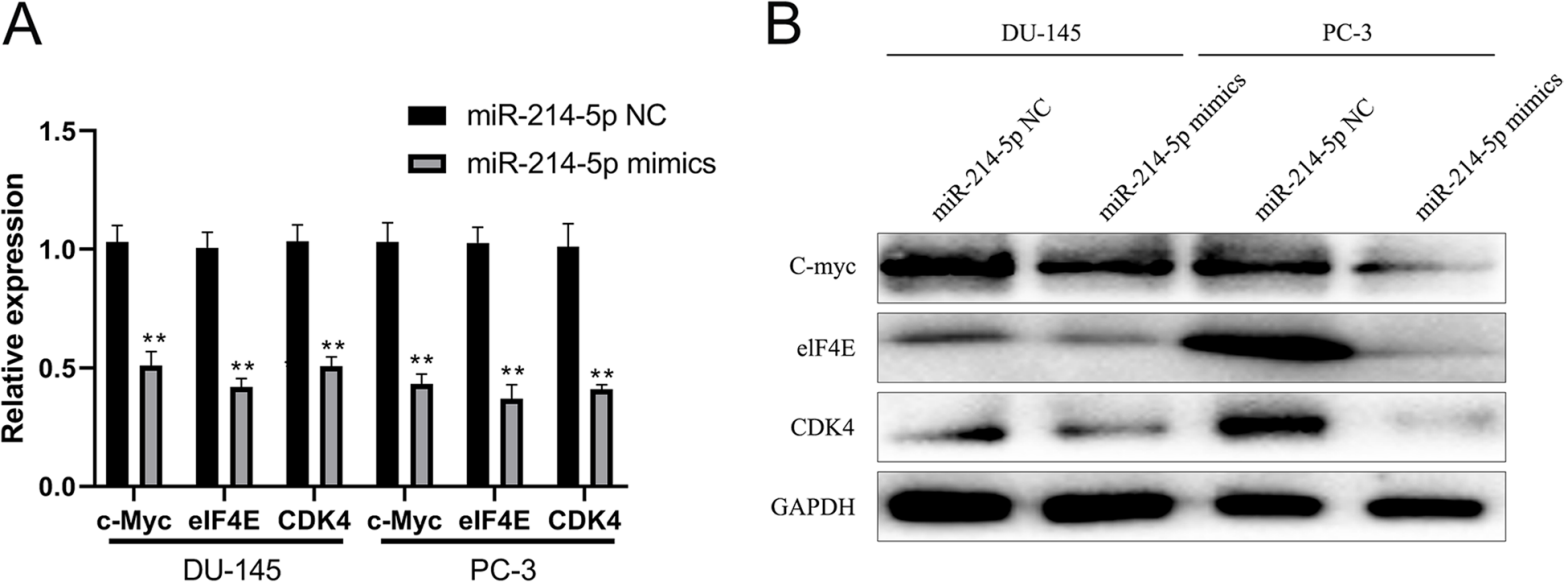
miRNA-214-5p inhibits the expression of downstream factors of SOX4 pathway. miRNA-214-5p mimics and negative control were transfected into DU-145 and PC-3 cells, respectively. Total RNA and protein were extracted 48 h later to detect the change of change of transcription and translation levels of c-Myc, eIF4E and CDK4 respectively with fluorescence quantitative PCR assay (**A**) and Western blot (**B**). miRNA control group was used as control (*P < 0.05, **P < 0.01)

According to the above results, lower expression of miRNA-214-5p was observed in prostate cancer patients, while higher expression of SOX4 was observed in prostate cancer patients. The overexpression of miRNA-214-5p significantly lowered the expression level of SOX4 gene in prostate cancer cells. In order to clarify the effect of miRNA-214-5p on SOX4 pathway, the change of transcription and translation levels of such SOX4 downstream factors as c-Myc, eIF4E, and CDK4 were detected. The results showed that c-myc, eIF4E, and CDK4 in miRNA-214-5p mimic transfection group were significantly lower than those in negative control transfection group regarding transcription (Fig. 5A) and translation level (Fig. 5B), indicating that miRNA-214-5p directly targeted SOX4 and affected the expression of downstream key growth factors.

### Specific knockdown of SOX4 inhibited cell proliferation

**Fig. 6:**
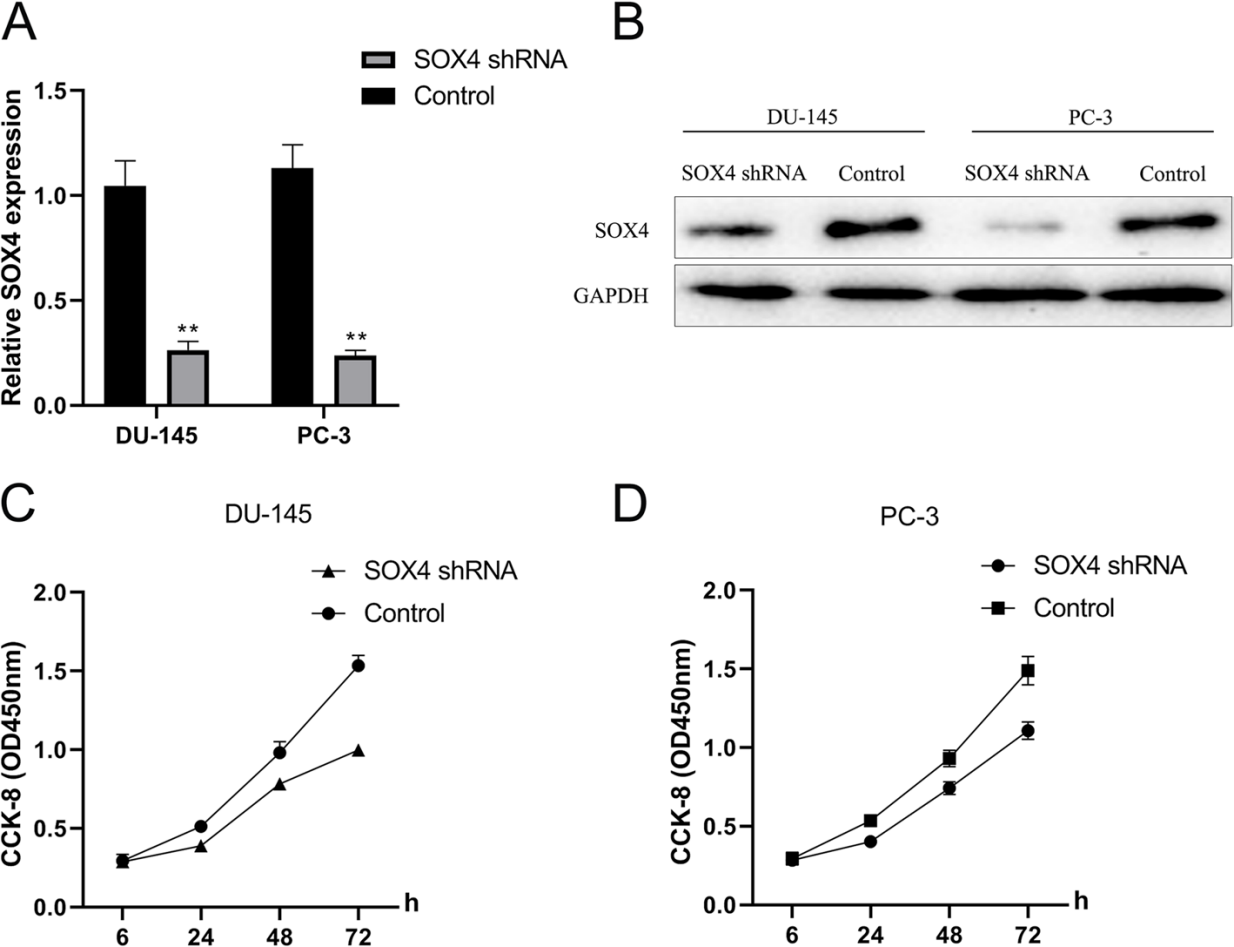
Specific knockdown of SOX4 significantly inhibits cell proliferation. Lentivirus-packaged SOX4 shRNA and control shRNA were transduced into DU-145 and PC-3 cells, respectively. The knockdown efficiency of shRNA at transcription and translation levels was verified by RT-qPCR (**A**) and Western blot (**B**). CCK-8 kit was used to detect the proliferation of SOX4 shRNA and control shRNA cell lines (**C**, **D**) at 6 h, 24 h, 48 h, and 72 h, respectively. GAPDH is an internal reference protein. miRNA control group was used as control (*P < 0.05, **P < 0.01)

The above results indicated that transfection of miRNA-214-5p mimics in prostate cancer cell lines DU-145 and PC-3 can significantly reduce the proliferation rate of cells and significantly downregulate the expression levels of SOX4 and its downstream key factors. In order to determine whether the inhibition of cell proliferation caused by overexpression of miRNA-214-5p was directly attributed to the change of SOX4 expression rather than other possible pathways, knockdown of SOX4 shRNA was performed into DU-145 and PC-3 cells through lentivirus transduction specificity, and the effect of SOX4 knockdown on cell proliferation was detected by CCK-8. The results showed that SOX4 specific knockdown (Fig. 6A, B) significantly inhibited cell proliferation (Fig. 6C, D), further indicating that miRNA-214-5p inhibited cell proliferation by targeting SOX4.

## Discussion

Prostate cancer is the most common malignant tumor in male cancer, and more than 160,000 new cases are diagnosed in the USA annually [32]. Although it is quite common, the painless course and the potential adverse effects of treatment of most prostate cancers have aroused discussions about screening and early detection. Nevertheless, we still cannot ignore the long-term impact of prostate cancer on men’s health. In the past 25 years, as great progress has been made in terms of the diagnosis and treatment of prostate cancer, the five-year survival rate of prostate cancer patients has been greatly improved. However, due to the lack of more sensitive and specific early diagnostic markers other than PSA, as well as specific drugs for the treatment of prostate cancer, if the tumor metastasis occurs, the mortality rate will be quite high. Therefore, prostate cancer remains the third leading cause of male cancer death around the world [33].

For the diagnosis of PSA, although it is widely used clinically, it has great limitations in finding cases, predicting the outcome of the disease, and guiding clinical management decision-making. For example, the specificity of PSA with a standard cutoff value of 4 ng/mL is 93.6%, but its sensitivity is only 20.5%, indicating that its sensitivity is extremely low [34]. Therefore, we urgently need to find new diagnostic markers to improve the accuracy of diagnosis, the accuracy of prognosis prediction, and the rationality of clinical treatment plans. Recent studies have shown that miRNAs play an important role in the development of prostate cancer. The results of these studies have shown that many miRNAs are expressed disorderly in prostate cancer patients’ tissues and suggested that certain specific miRNAs may grade prostate cancer. It has important guiding significance [35]. Related research results showed that miRNA-214-5p is abnormally expressed in prostate cancer cell lines. In view of this, this study analyzed the expression of miRNA-214-5p in clinically collected prostate cancer tissues, and the results showed that the expression level of miRNA-214-5p in prostate cancer tissues was significantly lower than that in non-cancer tissues, consistent with the results observed in cell lines (Fig. 2).

MicroRNAs (miRNAs) are 22-nucleotide non-coding RNA molecules that act as regulators of gene expression and have a series of biological functions such as regulating cell survival, proliferation, apoptosis, tumor growth, and metastasis [36]. MiRNAs bind to their complementary mRNA sequences, and ultimately lead to their translational inhibition, degradation, or cleavage. In the field of cancer research, researchers have found that the abnormal expression of miRNA may help the body’s immune system recognize cancer and non-cancer tissues. In recent years, more than 24,000 peer-reviewed articles and clinical studies have found that miRNAs play an important role in the field of cancer, and nearly 50% of miRNAs target sequences are located in cancer-related gene regions in the human genome [37]. Although several miRNAs have been discovered that can regulate the process of carcinogenesis, their clinical application has been limited because they cannot be accurately delivered to the tumor site, and because they have a wide range of functions and easily lead to off-target effects. Existing studies have shown that lentiviral vectors have effective cell delivery, but their oncogene activation and excessive immunogenicity have made researchers to worry about the safety of genome integration. In order to overcome these shortcomings, non-viral transmitters such as polyethyleneimine (PEI) nanoparticles, liposomes, polymer micelles, dendrimers, magnetic nanoparticles, and polymeric nanoparticles have been highly expected by researchers [38, 39]. These delivery mediators can protect miRNAs from intracellular nuclease degradation, increase their half-life in the blood-brain barrier, escape endosomal and lysosome degradation, and deliver miRNAs to the cytoplasm or nucleus to perform their biological functions. At the same time, in the research on prostate cancer, researchers found that miRNA-499a can inhibit the proliferation and apoptosis of prostate cancer cells [40], while miRNA-330-5p can effectively inhibit the development of prostate cancer [41]. These results showed that miRNAs have great potential in the treatment of prostate cancer.

In order to study the molecular mechanism of miRNA-214-5p involved in regulating prostate cancer, this study used bioinformatics methods to deeply analyze the host factors related to miRNA-214-5p in the prostate cancer database, and the results showed that miRNA-214-5p is specifically targeting the 3′-UTR region of the sex determining region Y-box 4 (SOX4) (Fig. 1). SOX4 is a developmental transcription factor, which plays an important role in the development of progenitor cells and Wnt signal transduction. It is necessary for precise differentiation and proliferation in a variety of tissues [42]. In addition, SOX4 is overexpressed in many human malignant tumors, but the mechanism of SOX4 in cancer progression is unclear. Although SOX2 is essential for the maintenance of stem cells, SOX4 may specifically express, transport, and expand progenitor cells. These progenitor cells are the direct progeny cells of adult stem cells and are considered to be the population that produces cancer stem cells. In humans, SOX4 is expressed in developing mammary glands and osteoblasts and is upregulated by progesterone. The expression of SOX4 showed an upregulation trend in prostate cancer cell lines and patient samples, and this upregulation was related to Gleason score or tumor grade [43]. In addition, SOX4 is overexpressed in many other types of human cancers, including leukemia, melanoma, glioblastoma, medulloblastoma, bladder cancer, and lung cancer [44]. A data analysis of human cancer transcription profiles found that SOX4 is one of 64 upregulated genes, which further indicates that SOX4 plays a role in many malignant tumors [42]. Although the research on SOX4 has been quite in-depth, its specific mechanism in the carcinogenic process remains to be explored. So far, researchers have used small interfering RNA knockdown, or tried to express SOX4 to identify its downstream target genes, and a chromosome immunoprecipitation experiment was carried out in liver cancer cells and identified 31 potential downstream genes of SOX4 [45], which laid a certain foundation for the follow-up related research. Through literature reports, we found that SOX4 may exist as an inhibitory regulator of many cancers.

Prostate cancer is closely related to the male population, one of the main characteristics of prostate cancer is its hormone responsiveness. This phenomenon was first discovered by Huggins and Hodges. They reported that castration miraculously led to tumor regression in prostate cancer patients [40]. Currently, the standard treatment for prostate cancer is Androgen deprivation therapy (ADT) using drugs that block the androgen pathway. However, with the extension of treatment, patients may develop resistance to ADT, leading to primary castrated prostate cancer (CRPC) or metastatic CRPC (mCRPC). In recent years, there has been an increase in the number of aggressive androgen receptor variant prostate cancers with neuroendocrine characteristics (NEPC) or small cell characteristics (small cell prostate cancer) with low expression or deletion. Some researchers believe that this may be related to the use of potent androgen receptor antagonists [41]. In addition, some androgen receptor-independent tumors do not express neuroendocrine differentiation markers. These variant cancers that are completely unresponsive to ADT treatment may come from rare and existing low-expressed or missing androgen receptor cancers or from normal cancers. Cancer cases that express androgen receptors have metastasized to low-expression or deletion mutation cases [46]. Based on the above and the preliminary results of this study, we speculate that SOX4 may have an important relationship with the pathogenesis of prostate cancer. The results of this study also showed that the expression of SOX4 in prostate cancer tissues was significantly higher than that in non-cancer tissues (Fig. 2). Further research results showed that inhibiting the expression of SOX4 in prostate cancer cell lines can inhibit the proliferation of cancer cells. Therefore, SOX4 may become the next potential clinical drug target for prostate cancer. Since the target of miRNA may not be unique, in addition to the host factor SOX4, miRNA-214-5p may have other target genes, and the combination of these genes is the key to the development of prostate cancer. Therefore, in follow-up research, we need more in-depth predictive analysis and miRNA-214-5p targeted genes. At the same time, the object used in this study is a cell line, which does not truly reflect the real situation in the body. Therefore, in the next study, we need to investigate whether SOX4 expression changes in the mouse model can inhibit the growth of prostate cancer tissue in vivo. At the same time, the SOX4 pathway growth-related factors c-Myc, eIF4E, and CDK4 will also undergo corresponding changes. Therefore, in the molecular diagnosis of prostate cancer, we can also introduce changes in the levels of c-Myc, eIF4E and CDK4 to evaluate the progression of prostate cancer.

The first miRNA alternative therapy entering clinical trials involves reconstitution of tumor suppressor miRNA (miR-34) in modified liposomes. MRX34 showed good results in a phase I clinical trial, and partial response was observed in patients with renal cell carcinoma, acral lentiginous melanoma, or hepatocellular carcinoma [47]. Many advanced patients showed good results in this trial. Therefore, the company will continue to advance this trial to phase II to obtain more reliable data to further pave the way for real clinical application. Recent studies have found that the growth rate and volume of bone tumor reduced by two times as chitosan-coated miR-34a was delivered to the body. It has been proved that exosomes can effectively deliver anti-miR-21 oligonucleotides to prostate cancer cells, significantly reducing the expression level of miR-21 and the motility of prostate cancer cells. miR-34a indicates the chemosensitization effect of paclitaxel on prostate cancer cells by targeting Bcl-2 protein. The combination of Let-7c miRNA with the nanoparticle-based system targeting prostate cancer cells showed more effective targeting and uptake [48]. At the same time, as the transmitter of miRNAs to tumor cells, the carrying capacity of gold nanoparticles is 10-20 times higher than that of liposomes. They also feature lower toxicity, more effective uptake capacity, faster endosome escape ability, and longer half-life [49].

Our results showed that miRNA-214-5p could specifically target the 3′UTR region of SOX4 gene, and this binding will reduce the expression of SOX4 and its downstream growth factors. In consideration of the actual process of drug delivery in vivo and the action time, the development of these new miRNA transmitters will be more favorable for miRNA-214-5p to target SOX4 gene. At the same time, the longer half-life and immune escape ability will bring miRNA-214-5p into full play. In conclusion, the results of these in vivo experiments will provide certain basis for the clinical trials of miRNA-214-5p in the future. At the same time, the development of new media will also provide a strong support for the efficient targeting of miRNA-214-5p.

According to the results of this experiment in vitro, miRNA-214-5p may also inhibit the proliferation of prostate cancer cells in vivo. However, continuous study should be carried out and efforts should be made to verify the role of miRNA-214-5p in mouse models. In view of the role of SOX4 in prostate cancer, if we try to inhibit the expression of SOX4 in vivo, it may also alleviate the development of prostate cancer. Combined with miRNA therapy, it will further improve the therapeutic effect of prostate cancer.

## Conclusion

miRNA-214-5 can inhibit the proliferation of prostate cancer cells by specifically targeting S0X4 and inhibiting the expression of growth factors downstream of this pathway.

## Data Availability

The datasets used and/or analyzed during the current study are available from the corresponding author on reasonable request.
